# Artesunate alleviates the inflammatory response of ulcerative colitis by regulating the expression of miR-155

**DOI:** 10.1080/13880209.2020.1867196

**Published:** 2021-02-01

**Authors:** Zhao-Bin Yang, Lu-Zhen Qiu, Quan Chen, Jian-Dong Lin

**Affiliations:** a Department of Medical Intensive Care Unit, Zhangzhou Affiliated Hospital of Fujian Medical University, Zhangzhou, Fujian, P. R China; b Department of Neurosurgery, Fujian Medical University Union Hospital, Fuzhou, Fujian, P. R China; c Department of Intensive Care Unit, The First Affiliated Hospital of Fujian Medical University, Fuzhou, Fujian, P. R China

**Keywords:** NF-κB, microRNA, natural plant extracts, cellular molecular mechanism

## Abstract

**Context:**

Ulcerative colitis (UC) is a recrudescent and chronic inflammatory disease. Artesunate (ART) has shown its anti-inflammatory and antioxidative properties in severe diseases, including UC.

**Objective:**

The present study investigates the molecular mechanisms for effects of ART on UC, and the role of miR-155 in this process.

**Materials and methods:**

The *in vitro* UC model was established by using lipopolysaccharide (LPS)-induced RAW264.7 cells. For BALB/c mice model, different concentrations/doses of ART were treated once a day for 7 days. The apoptosis and viability were measured by CCK-8 and flow cytometry assay, respectively. The expressions and concentrations of inflammatory factors were detected by qRT-PCR and ELISA, respectively. Colon tissues of mice were used for detecting the activity of MPO, and the histological changes were observed by H&E staining.

**Results:**

The IC_50_ of ART for RAW264.7 cells was 107.3 μg/mL. In LPS-induced cells, ART treatment inhibited the cell apoptosis and promoted cell viability compared with the model group. Besides, ART treatment also reduced the expressions of pro-inflammatory factors and miR-155. However, overexpression of miR-155 showed opposite effects and attenuated the effects of ART. Meanwhile, inhibiting miR-155 expression also improved the inflammatory response induced by LPS. In UC mice model, ART treatment also alleviated the mice’s survival and alleviated the inflammatory response. In addition, the expression of p-NF-κB was suppressed by ART.

**Conclusion:**

ART reduced the inflammatory response by inhibiting the expression of miR-155 in UC to inhibit the NF-κB pathway. This research showed ART might have potential in UC treatment.

## Introduction

Ulcerative colitis (UC) is an idiopathic and extensive chronic inflammatory disease, mainly affecting the colon (Xue et al. 2018). UC is a major risk factor for the development of colorectal cancer, which is characterised by diffuse mucosal inflammation of the colon (Kim et al. 2009; Xue et al. 2018). Although the exact pathogenic mechanism of UC development is not yet known, the genetic predisposition and geographical or environmental conditions are believed to be involved. Additionally, evidence suggests that increased permeability, as well as infiltration of inflammatory cells by an angiogenic factor VEGF may as well contribute to UC (Shanahan 1993; Tolstanova et al. 2010).

Artesunate (ART) is a water-soluble semisynthetic derivative of the sesquiterpene lactone compound artemisinin (Li et al. 2019). It has been used to treat severe malaria with desirable outcome for a number of years. Most importantly, it is considered to be safe with minimal side effects than quinine (a traditionally prescribed drug to treat severe malaria) (Dondorp et al. 2010; Kunte and Kunwar 2011). Studies have also shown that it has activities other than being antimalarial, such as antitumor, anti-inflammatory, as well as antioxidative properties (Zhao and Song 1989; Zuo et al. 2016). In a study aimed at evaluating the role of ART and its possible mechanism of action in DSS-induced colitis, it was reported that ART alleviated UC via down-regulation of inflammatory and apoptotic markers by regulating the TLR4/nuclear factor (NF)-κB signalling pathway (Chen et al. 2019), suggesting that ART has anti-inflammatory properties. However, how it suppresses inflammatory responses at the molecular level still needs to elucidate.

MicroRNAs (miRNAs) are short strands of non-coding single-stranded RNA molecules of approximately 22 nucleotides in length encoded by endogenous genes that regulate post transcription expression (Kalla et al. 2015). Studies have shown a more significant role for miRNAs in a number of diseases including UC (Tian et al. 2016; Xu and Zhang 2016). For example, miR-155 is known to be up-regulated in UC where it induces intestinal inflammation through up-regulation of Th1 and Th17 responses (Singh et al. 2014). Additionally, (Béres et al. 2016) reported elevation of miR-146a and miR-155 in UC and Crohn’s disease (CD) patients. Other studies have also shown that miR-155 is significantly up-regulated in blood samples from UC patients and is the highest among all the up-regulated miRNAs (Min et al. 2014; Singh et al. 2014). However, the relationship between ART and miR-155 in UC has not been illuminate yet.

We herein report that ART attenuated the pro-inflammatory response in UC via the NF-κB signalling pathway through suppression of miR-155. These findings might give deeper insights for ART in UC treatment and provide some new potential research target for molecular mechanisms of UC development.

## Materials and methods

### Cell culture and treatment

RAW264.7 cells were purchased from the Cell Bank of the Chinese Academy of Science (Shanghai, China). Cells were cultured in Dulbecco’s modified Eagle’s medium (DMEM, Gibco BRL, Rockville, USA) containing 10% (v/v) foetal bovine serum (FBS, Gibco), 100 units/mL penicillin and 100 μg/mL streptomycin at 37 °C in a humidified incubator with 5% CO_2_ and 95% sterile air.

**Figure 1.**
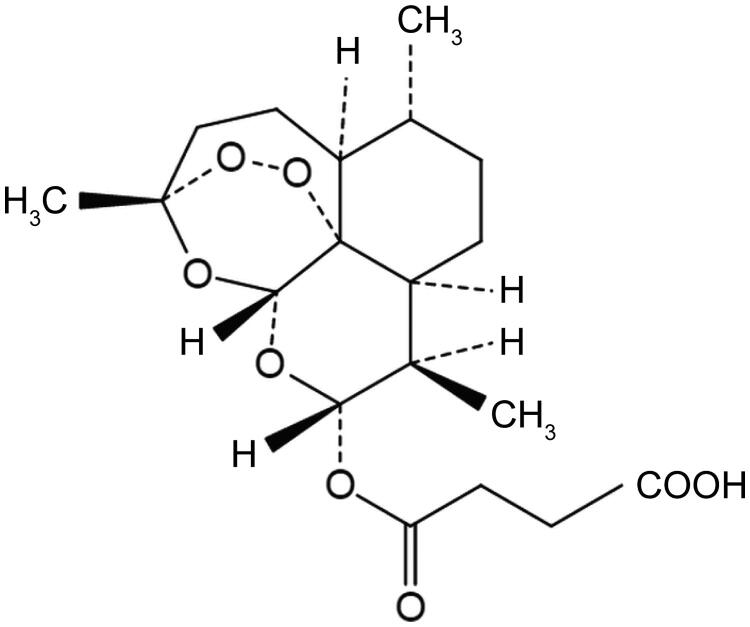
Chemical structure of artesunate (ART).

After reaching 80%–85% confluence, cells were digested by trypsin enzyme, plated in 24-well plates (1 × 10^5^ cells/well) and incubated at 37 °C for 24 h. Subsequently, RAW264.7 cells were stimulated with 1 μg/mL lipopolysaccharide (LPS) (Sigma-Aldrich, St. Louis, MO, USA) for 48 h to construct an UC model. Then cells were treated for 24 h with different concentrations of ART (Sigma-Aldrich, St. Louis, MO, USA), the low concentration (LC, 5 μg/mL), the middle concentration (MC, 10 μg/mL) and the high concentration (HC, 20 μg/mL), respectively. The chemical structure of ART is shown in Figure 1.

### Cell transfection

For cell transfection, the cells were transfected with 100 nM of the miR-155 mimics or negative control (NC) (all synthesised and purchased from GeneCopoeia, Guangzhou, China) by using Lipofectamine 2000 (Invitrogen) following the manufacture’s protocol.

### Cell counting kit-8 (CCK-8) assay

The viability of RAW264.7 cells was measured by using the CCK-8 method. Cells (5 × 10^3^ cells/well) were plated in 96-well plates and incubated at 37 °C for 4 h. CCK-8 reagent was then added to the 96-well plates, and the cells were further cultured at 37 °C, 5% CO_2_ for 1 h. Absorbance was detected at 490 nm by using a microplate reader (Bio‑Rad, Hercules, CA, USA).

### 3-(4,5-Dimethylthiazol-2-yl)-2,5-diphenyltetrazolium bromide (MTT) assay

For MTT assay, cells with density of 3 × 10^4^ were cultured in 96-well plates for 48 h at 37 °C and 5% CO_2_. After addition of 10 μL MTT solution (5 mg/mL), the cells were subsequently cultured for 4 h and the MTT was removed. Then 200 μL DMSO was added and the value of optical density (OD) was evaluated 490 nM.

### Flow cytometry assay

The apoptosis of RAW264.7 cells was analysed using the flow cytometry assay. The RAW264.7 cells (1 × 10^5^ cells/mL) were collected in a 15 mL tube after treatment. Following staining with Annexin V-APC and propidium iodide (PI) reagent (KeyGen BioTech, China) for 15 min at room temperature in dark and the cell apoptosis was rapidly analysed using a FSCAN flow cytometer (BD Biosciences, USA).

### Animals and treatment

Male BALB/c mice (4- to 6-week-old; 18–20 g) used in this study were purchased from Beijing Vital River Laboratory Animal Technology Co., Ltd (Beijing, China). The mice were housed at 24 ± 2 °C and 50–60% relative humidity in a SPF environment, and kept on 12 h light/darkness cycle. Food and water were provided *ad libitum*. The experimental protocols followed the National Institutes of Health guide on animal handling and were approved by the Fujian Medical University Animal Experiment Ethics Committee prior to commencement of the study.

The mouse model of UC was induced by using TNBS (2.5%)/ethanol (50%) complex method as reported elsewhere (Liao et al. 2016). Briefly, 0.1 mL of 2.5% TNBS (dissolved in 50% ethanol) was slowly instilled into the mice’ anus, then the mice were kept vertical for 1 min. The control mice were treated with 50% ethanol by the same operation. For treatment of ART, the mice were slowly injected with different concentrations/doses by gavage administration [low concentration (LC) 50 mg/kg, middle concentration (MC) 100 mg/kg and high concentration (HC) 150 mg/kg] (Yang et al. 2012) of ART dissolved in 0.2 mL saline into the peritoneum after 8 h of TNBS treatment. The mice were randomly divided into five groups of 20 mice per group: the ethanol control group (Control), TNBS model group (TNBS), TNBS + LC group, TNBS + MC group and TNBS + HC group. The control and TNBS groups were injected with 0.2 mL saline. All mice were injected once per day for 7 days, and were then euthanized for the next experiments.

### Colon morphology

The colons were immediately resected after sacrificing the mice. The fat and mesenteric tissues were discreetly removed, then the colon length was measured using graduated scale. The intestine of colon was washed with PBS, the distal colon (∼1 cm) was fixed in 10% formalin, and embedded in paraffin. Then samples were cut to 5 mm sections and placed on the slides. Subsequently, hematoxylin-eosin (H&E) staining was performed to observe the histological changes using an optical microscope (Olympus, Japan). The histological score of colon’s H&E slides were evaluated in accordance with the angles of ulcer formation, colonic crypt damage, mucosal erosion and lymphocyte infiltration following the classic scoring system in a blinded manner (Cooper et al. 1993).

### Detection of myeloperoxidase (MPO) in Colon tissue

Briefly, the colon tissues (1 g) were collected, homogenated in 2 mL PBS and the supernatants were isolated by centrifugation at 10,000 rpm for 10 min at 4 °C. Then, the activity of MPO was measured using an MPO assay kit (Abcam, Cambridge, MA, USA) according to the manufacturer’s instruction.

### Real-time quantitative PCR detection (qRT-PCR)

The mRNA levels of miR-155 and inflammatory factors interleukin (IL)-12, IL-23, IL-17 and tumour necrosis factor (TNF)-ɑ were detected by qRT-PCR. Briefly, total RNA was extracted from RAW264.7 cells and mouse colon tissues by using TRIzol regent (Invitrogen). RNA was then reverse transcribed into cDNA using the PrimeScript RT reagent Kit (Invitrogen) according to the manufacturer’s procedure. The resultant cDNA was stored at −20 °C for subsequent qRT-PCR reactions which were performed by SYBR-Green Master Mix (Thermo). The qRT-PCR amplification (10 μL) conditions were as follows: At 95 °C for 30 s, followed by 40 cycles of 95 °C for 15 s, 60 °C for 30 s. The fold relative expression of target gene was analysed according to the 2^−ΔΔCt^ method (Livak and Schmittgen 2001), and standardised to GAPDH. QRT‑PCR primers used in this paper are presented in Table 1.

**Table 1.** QRT‑PCR primer sequences in this study.

Gene name	Forward primer (5′→3′)	Reverse primer (5′→3′)
miR-155	AGAGAGCTCAGATTTGACCGGCAGCGCCC	GAGATCTAGATGCACAGCAGAAAAATAAAGCCAGA
IL-12	ATGACCCTGTGCCTTGGTAG	TCTCCCACAGGAGGTTTCTG
IL-17	TCCCTCTGTGATCTGGGAAG	AGCATCTTCTCGACCCTGAA
IL-23	CCAGCGGGACATATGAATCT	CATGGGGCTATCAGGGAGTA
TNF-α	CCGATGGGTTGTACCTTGTC	TGGAAGACTCCTCCCAGGTA
U6	CGCAAGGATGACACGCAAAT	GTGCAGGGTCCGAGGTATTC
GAPDH	AGCCCAAGATGCCCTTCAGT	CCGTGTTCCTACCCCCAATG

### Enzyme linked immunosorbent assay (ELISA)

The culture supernatants of RAW264.7 cells were collected. Blood samples were obtained from the tail veins of mice at 24 h after final administration. The blood was then centrifuged at 1000 rpm for 5 min. The levels of inflammatory factors (IL-12, IL-23, IL-17 and TNF-ɑ) in either cell supernatants or mouse serum samples were measured by ELISA kits (Abcam) according to the manufacturer’s instructions.

### Western blotting

For western blotting, total proteins were extracted from the RAW264.7 cells or mice’s colon tissue by using a protein isolation kit (ThermoFisher Scientific, Waltham, MA, USA). The proteins were separated by 10% sodium dodecyl sulphate polyacrylamide gel electrophoresis (SDS-PAGE), and transferred onto polyvinylidene difluoride (PVDF) membranes. After blocking in 5% skim milk for 2 h at room temperature, the samples were incubated with primary antibodies (all purchased from Abcam) of NF‑κB (ab32360, 1/500), p-NF-κB (ab194729, 1/500) and β-actin (ab5694, 1/500) at 4 °C overnight. Subsequently, the membranes were washed with TBST, and then incubated with horseradish peroxidase‑conjugated goat anti‑rabbit IgG (ab6721, 1/500) for 2 h at room temperature. Then, the bands were developed with ECL (Beyotime Institute of Biotechnology) and were analysed by ImageJ software.

### Statistical analysis

All experiments were repeated at least 3 times, and the data were presented as mean ± SD. Statistical differences among three or more groups were analysed by using one-way analysis of variance (ANOVA) followed by Tukey *post hoc* test using SPSS software (SPSS, version 19.0; SPSS Inc., Chicago, USA). *P* < 0.05 was considered to be statistically significant difference.

## Results

### ART decreased expression of miR-155, enhanced cell viability and inhibited cell apoptosis in LPS-induced RAW264.7 cells

**Figure 2.**
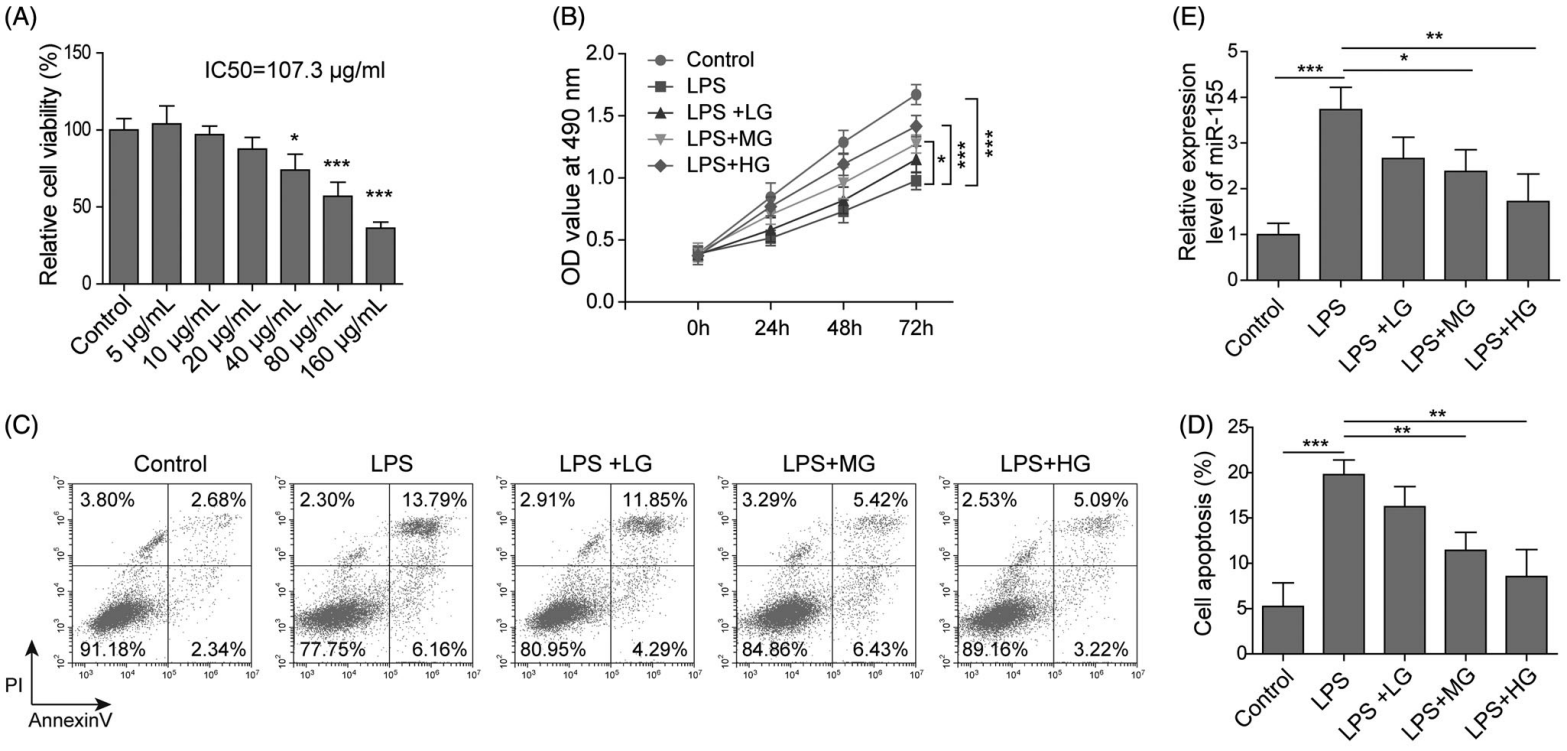
Viability, apoptosis and expression of miR-155 in UC cells after ART treatment. (A) The viability of cells by different concentrations of ART treatment (0, 5, 10, 20, 40, 80 and 160 μg/mL) was assessed by MTT assay. (B) The viability of cells was measured CCK-8 assay. (C, D) The apoptosis of cells was measured by flow cytometry. (E) The expression level of miR-155 was detected using qRT-PCR. Data were presented as mean ± SD. ns: not significant; *p < 0.05; **p < 0.01; ***p < 0.001.

To investigate the role of ART in UC, we used different concentrations of ART to treat the LPS-induced RAW264.7 cells. Firstly, MTT assay was used to measure the cell viability after different concentrations of ART treatment (0, 5, 10, 20, 40, 80, and 160 μg/mL). It was found the cell viability did not change significantly under 0, 5, 10 and 20 μg/mL ART treatment (Figure 2(A)). However, when treated by 40 ∼ 160 μg/mL ART, the cell viability reduced remarkably in a dose-dependent manner. The IC_50_ value for ART was 107.3 μg/mL (Figure 2(A)). As shown in Figure 2(B), the viability of RAW264.7 cells was significantly decreased after LPS treatment and was remarkably enhanced by treatment of all concentrations of ART in a dose-dependent manner. On the contrary, ART treatment markedly decreased the LPS-induced cell apoptosis, which was also in a dose-dependent manner (Figure 2(C,D)). Meanwhile, the LPS-induced expression of miR-155 was reduced by treatment of ART in a dose-dependent manner (Figure 2(E)). All these results suggested that ART could enhance the cell viability and suppress cell apoptosis in the LPS-induced UC *in vitro* model.

### ART decreased levels of LPS-induced pro-inflammatory factors

**Figure 3.**
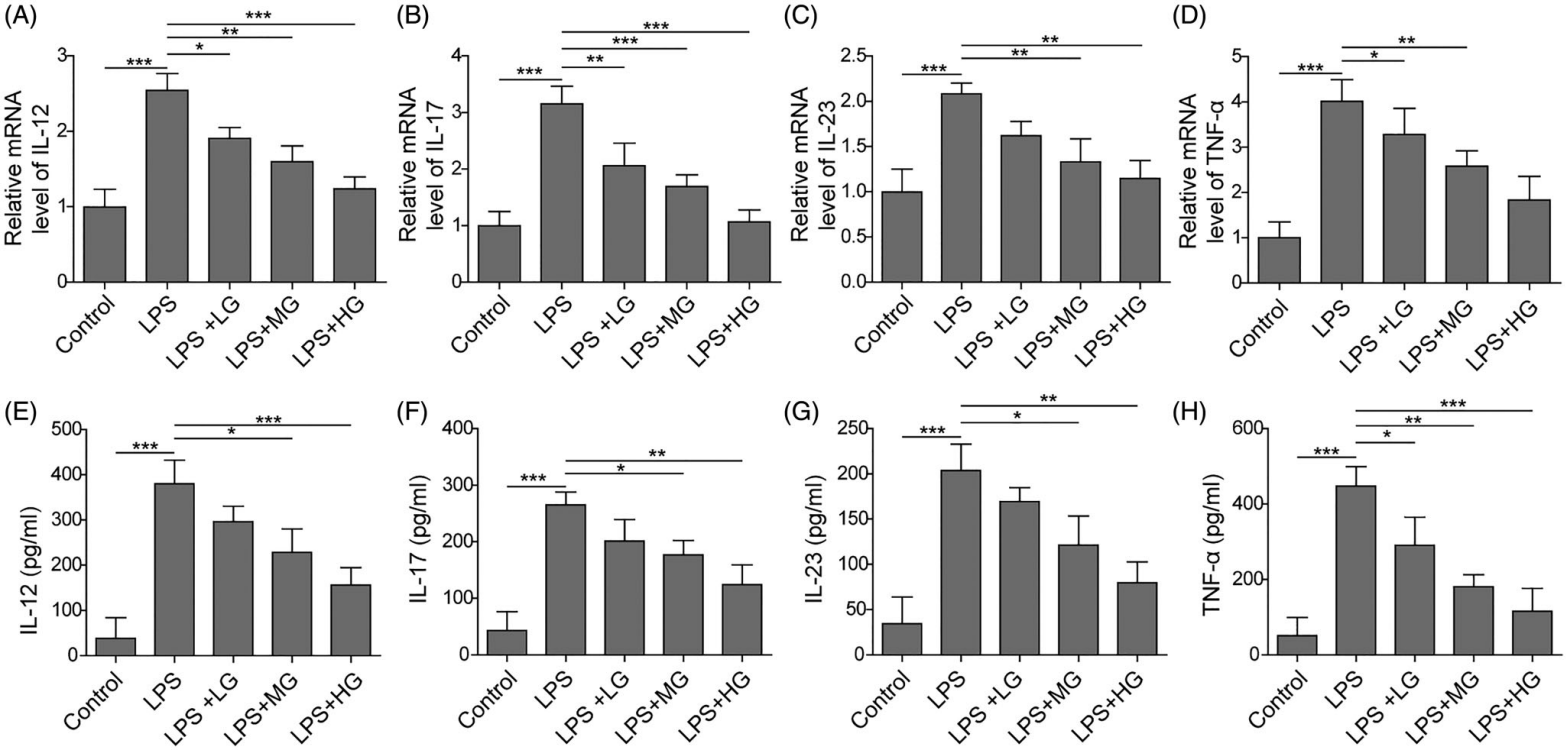
ART were down-regulated the expression of pro-inflammatory factors. The expression of IL-12 (A), IL-17 (B), IL-23 (C) and TNF-ɑ (D) were detected by qRT-PCR. The concentrations of IL-12 (E), IL-17 (F), IL-23 (G) and TNF-ɑ (H) in culture supernatants were detected using ELISA assay. Data were presented as mean ± SD. ns: not significant; *p < 0.05; **p < 0.01; ***p < 0.001.

Subsequently, the mRNA levels and cell supernatant concentrations of IL-12, IL-17, IL-23 and TNF-α in LPS-induced RAW264.7 cells were detected by qRT-PCR and ELISA methods, respectively. It was found that the mRNA levels of pro-inflammatory factors of IL-12, IL-17, IL-23 and TNF-α were remarkably increased after LPS treatment, which were significantly suppressed by treatment of ART in a dose-dependent manner (Figure 3(A–D)). Similar results were also found for the above inflammatory factors in cell supernatant concentrations (Figure 3(E–H)). These results indicated that ART reduced the levels of pro-inflammatory factors.

### MiR-155 reversed the inhibitory effects of ART on cell viability in LPS-induced RAW264.7 cells

**Figure 4.**
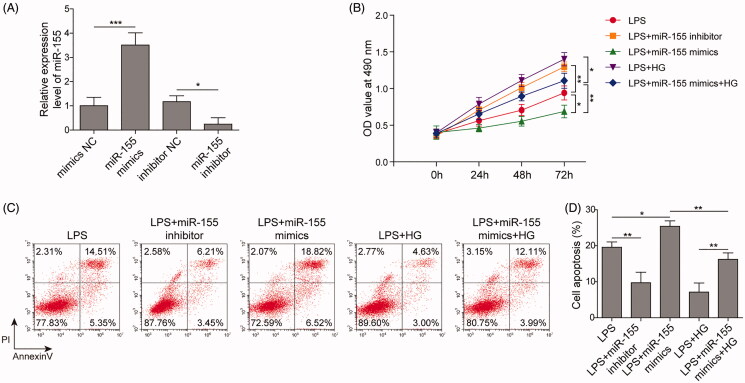
MiR-155 attenuated the effect of ART on cell viability. (A) The expression of miR-155 in RAW264.7 cells after transfected with miR-155 mimics and miR-155 inhibitor were detected by qRT-PCR. (B) The viability of cells was assayed by CCK-8. (C,D) The apoptosis of cells was measured by flow cytometry. Data were presented as mean ± SD. ns: not significant; *p < 0.05; **p < 0.01; ***p < 0.001.

To further demonstrate the role of miR-155 in UC development, miR-155 was overexpressed and inhibited by transfection of miR-155 mimics and inhibitor respectively. As shown in (Figure 4(A)), the expression of miR-155 in RAW264.7 cells was markedly increased and decreased after transfection with miR-155 mimics and inhibitor, respectively indicating the successful transfection. Cell viability was remarkably decreased by transfection with miR-155 mimics, but increased by miR-155 inhibitor. Besides, when miR-155 was overexpressed, the cell viability, which was enhanced by the high concentration of ART, was significantly reduced in LPS-induced cells (Figure 4(B)). Similarly, the cell apoptosis was promoted by overexpression of miR-155, while inhibition of miR-155 inhibited apoptosis. And the overexpression of miR-155 remarkably enhanced the cell apoptosis, which was inhibited by ART treatment (Figure 4(C,D)), implying miR-155 was able to attenuate the effects of ART on cell viability and apoptosis in LPS-induced RAW264.7 cells.

### MiR-155 reversed anti-inflammatory effects of ART in LPS-induced RAW264.7 cells through NF-κB signalling

**Figure 5.**
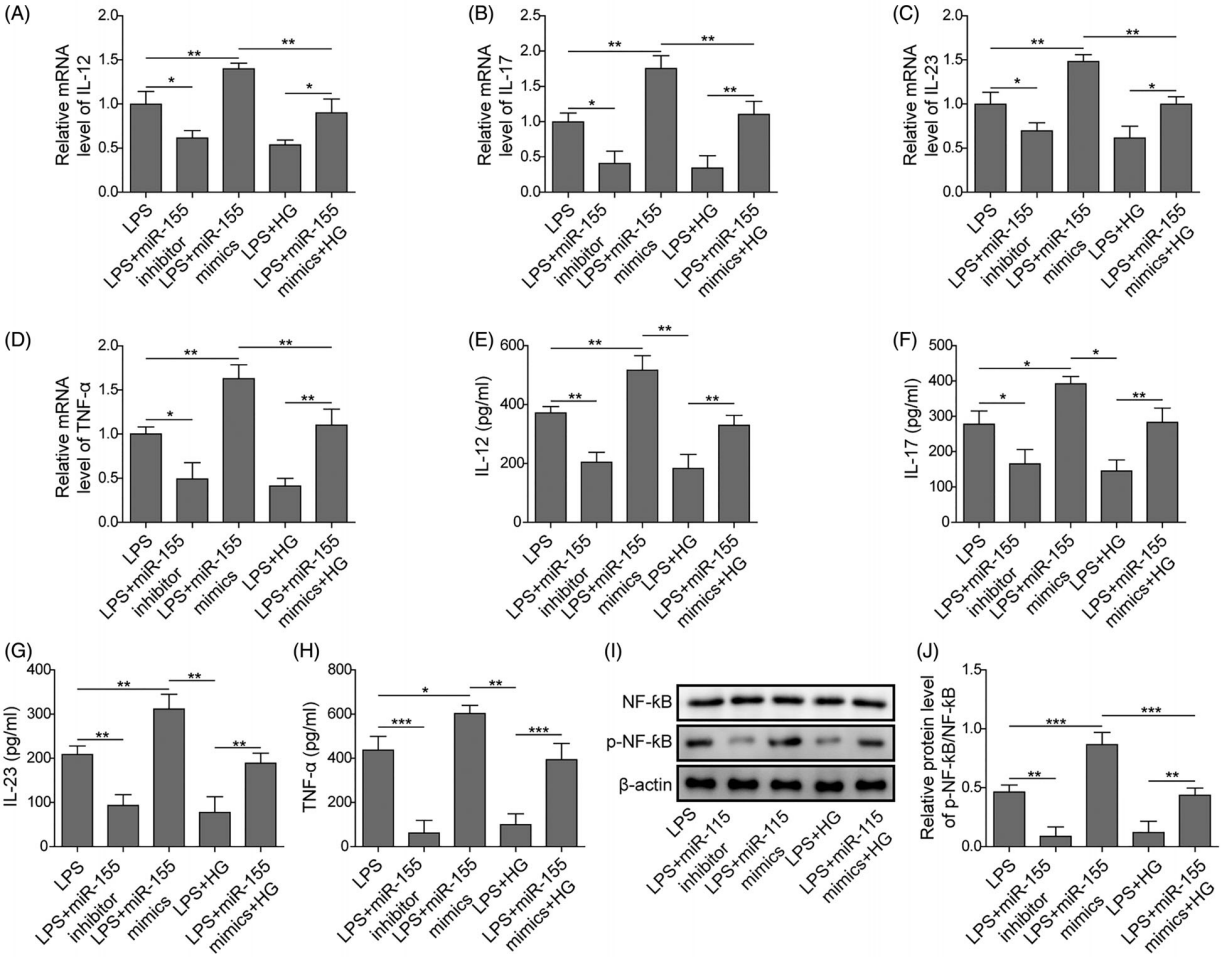
MiR-155 attenuated the regulation of ART on inflammatory factors, and regulated NF-κB signalling pathway *in vitro*. The expression of IL-12 (A), IL-17 (B), IL-23 (C) and TNF-ɑ (D) were detected by qRT-PCR. The concentrations of IL-12 (E), IL-17 (F), IL-23 (G) and TNF-ɑ (H) in culture supernatants were detected using ELISA assay. (I, J) The NF-κB signalling was detected by western blot. Data were presented as mean ± SD. ns: not significant; *p < 0.05; **p < 0.01; ***p < 0.001.

Then, we further measured the levels of inflammatory factors as well as detected the NF-κB signalling pathway when miR-155 was overexpressed. For mRNA levels, the overexpression of miR-155 significantly enhanced the levels of LPS-induced pro-inflammatory factors IL-12, IL-17, IL-23 and TNF-α, while inhibiting miR-155 could reduce pro-inflammatory factors expression (Figure 5(A–D)). Similarly, the cell supernatant levels of the pro-inflammatory factors were also increased by overexpression of miR-155, which was inhibited by transfection of miR-155 inhibitor (Figure 5(E–H)). Furthermore, the overexpression of miR-155 markedly reversed the effects of ART on the above inflammatory factors. Additionally, we measured the NF-κB signalling related proteins and found overexpression of miR-155 significantly increased the protein levels of p-NF-κB, whose total protein level did not change significantly. The protein levels of p-NF-κB were remarkably decreased by treatment of ART or inhibition of miR-155. However, overexpression of miR-155 dramatically reversed the effects of ART on NF-κB signalling (Figure 5(I,J)). All these results suggested that ART treatment might suppress the LPS-induced inflammatory effects through regulation of miR-155/NF-κB axis.

### ART alleviated inflammatory response in TNBS-induced UC by reducing miR-155 expression in vivo

**Figure 6.**
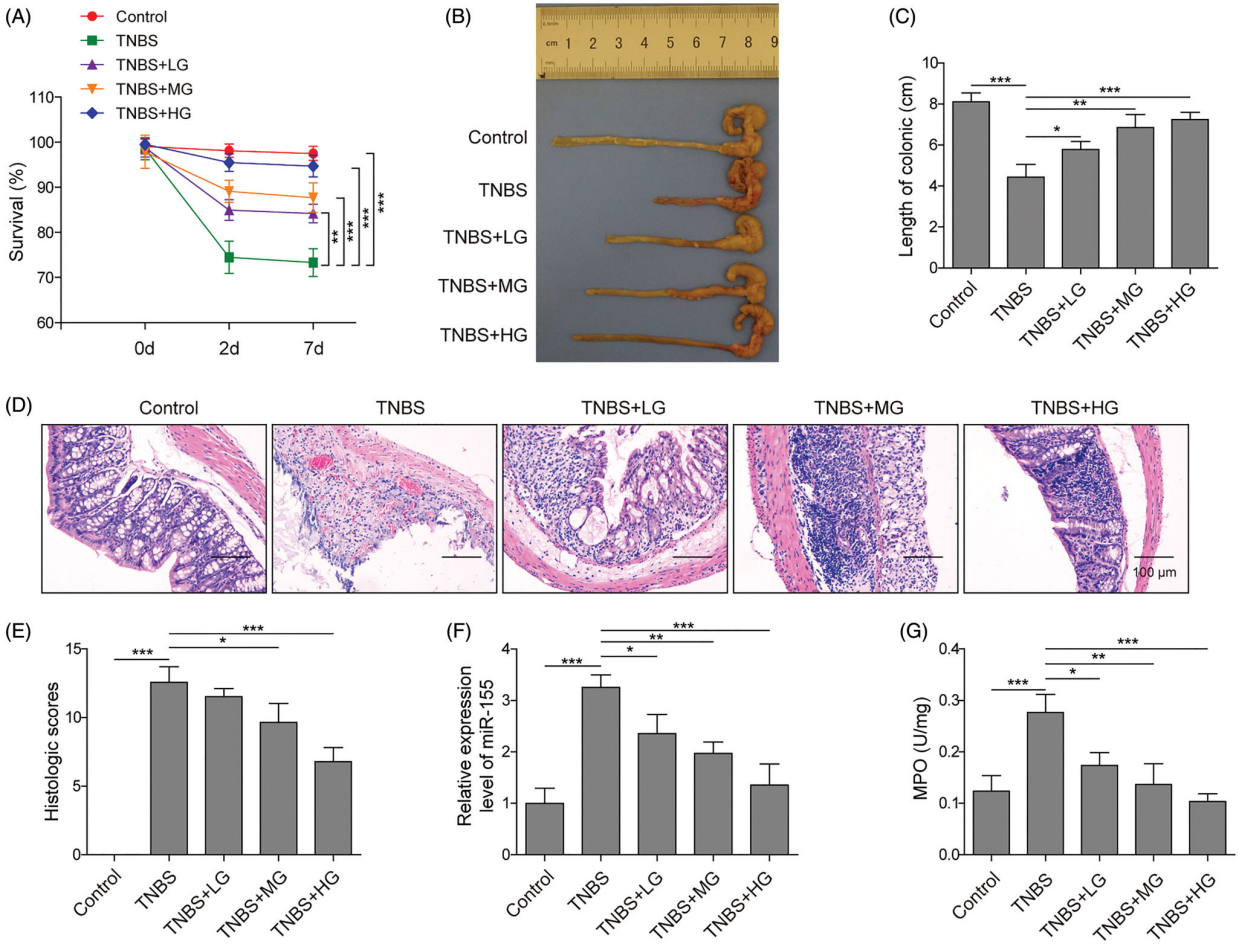
ART alleviated inflammatory response in mice with UC by reducing the expression of miR-155. (A) Mice survival rate during the ART treatment. (B, C) Colon length of mice. (D) H&E staining of colon tissues. (E) Histological score of colon tissues after H&E staining. (F) The expression of miR-155 was detected by qRT-PCR. (G) The activity of MPO. Data were presented as mean ± SD. ns: not significant; *p < 0.05; **p < 0.01; ***p < 0.001.

To further evaluate the effects of ART on UC, we treated TNBS-induced mice with different concentrations of ART. As shown in Figure 6(A), ART treatment significantly improved the survival rate of the mice, and mice treated with higher concentration of ART showed remarkably higher survival rate. It was also observed that ART treatment markedly increased the colon length of TNBS-induced mice in a dose-dependent manner (Figure 6(B,C)). Further histological analysis showed ART significantly improved the TNBS-induced tissue injury and reduced ulceration in colon tissues (Figure 6(D,E)). We also found miR-155 was significantly up-regulated in TNBS-induced mice and ART remarkably reduced its expression (Figure 6(F)). Besides, ART also decreased the TNBS-induced MPO activity (Figure 6(G)). All these results were in a dose dependent manner. These results suggested that ART alleviated inflammatory response in mice with UC by down-regulating the expression of miR-155.

### ART attenuated LPS-induced inflammatory effects through inhibition of NF-κB signalling via suppressing miR-155

**Figure 7.**
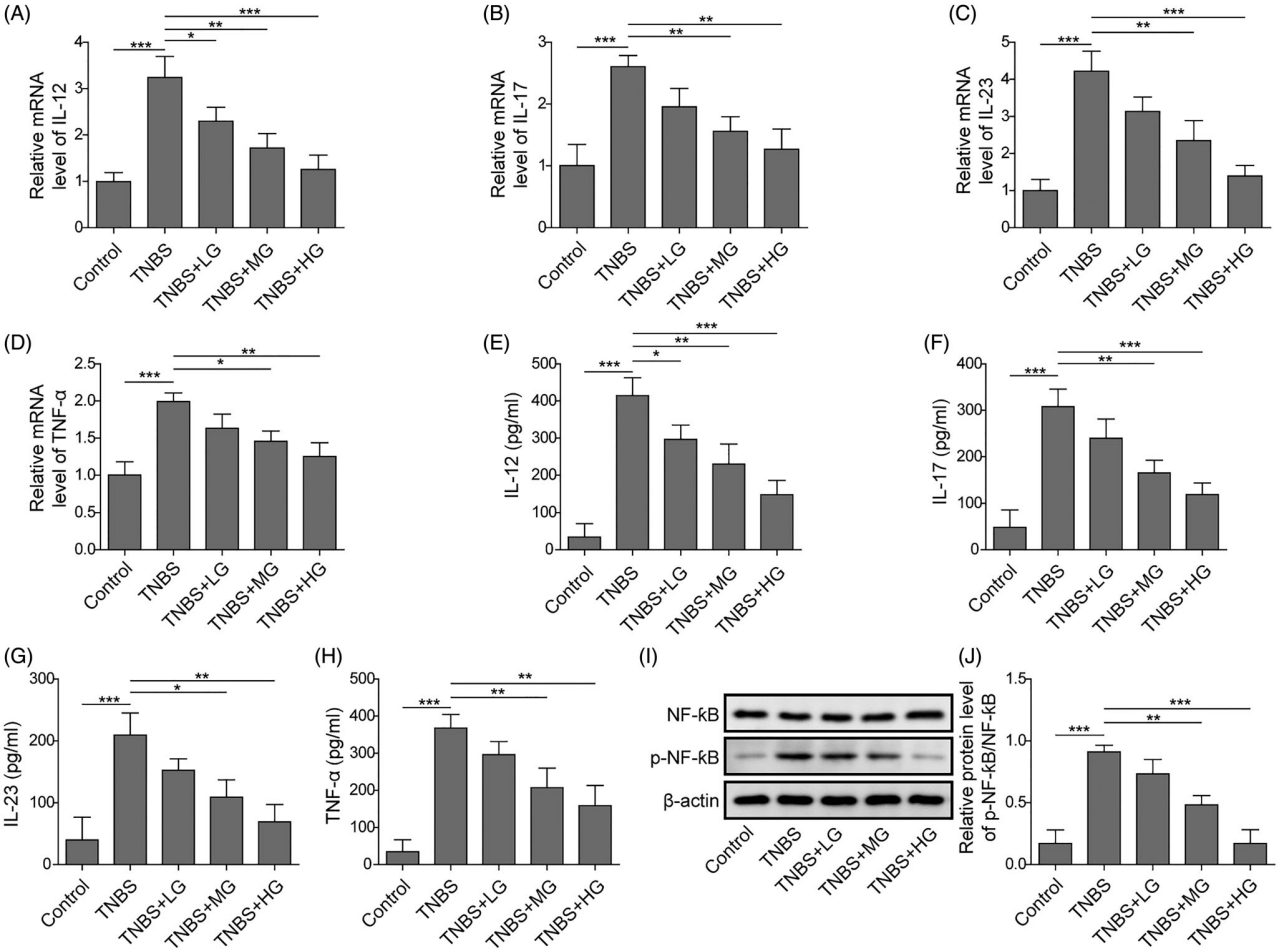
ART regulated the expression of inflammatory factors by reducing miR-155 expression, and inhibits NF-κB signalling pathway in the mouse model. The expression of IL-12 (A), IL-17 (B), IL-23 (C) and TNF-ɑ (D) were detected by qRT-PCR. The concentrations of IL-12 (E), IL-17 (F), IL-23 (G) and TNF-ɑ (H) in culture supernatants were detected using ELISA assay. (H, J) NF-κB signalling was detected by Western blot. Data were presented as mean ± SD. ns: not significant; *p < 0.05; **p < 0.01; ***p < 0.001.

Finally, we analysed the levels of inflammatory factors in colon tissues of TNBS-induced mice, as well as detected the NF-κB signalling pathway. Similar to the *in vitro* results, for both mRNA and serum levels, TNBS treatment significantly increased the levels of pro-inflammatory factors (IL-12, IL-17, IL-23 and TNF-α) (Figure 7(A–H)). However, treatment of ART remarkably suppressed TNBS induced activation of inflammatory response in a dose-dependent manner. The protein levels of p-NF-κB were also promoted by TNBS. And treatment of ART remarkably suppressed the TNBS-induced activation of NF-κB signalling in a dose-dependent manner (Figure 7(I,J)). All the findings suggested that ART may regulate the expression of inflammatory factors by down-regulating miR-155 inhibiting the activation of NF-κB signalling pathways *in vivo*.

## Discussion

UC is a recrudescent disease caused by inflammatory response of the gastrointestinal system (Głąbska et al. 2019). In recent years, the morbidity of UC has gradually increased, forcing the scientific community to devise new treatment methods (da Silva et al. 2015; Kim et al. 2017; Zhong et al. 2017; Castelli et al. 2018; Głąbska et al. 2019). However, despite numerous efforts, the treatment of UC is still a clinical challenge. In the present study, we demonstrated for the first time that ART could alleviate the inflammatory response of UC by downregulating the expression of miR-155 in both *in vitro* and *in vivo* models.

The anti-inflammatory effects of ART, as well as its function in immune diseases has been noticed in recent years (Dondorp et al. 2010; Li et al. 2013). It was found ART had immunosuppressive activity both *in vitro* and *in vivo*, and could inhibit T lymphocyte proliferation induced by mitogen and alloantigen (Li et al. 2013). Another study found ART could attenuate LPS-induced osteoclastogenesis by suppressing TLR4/TRAF6 and PLCγ1-Ca2 + -NFATc1 signalling (Zeng et al. 2019). In a recent research, it was observed that ART improved cerebral ischemia‑reperfusion injury through suppressing oxidative and inflammatory processes by activating Nrf2 and ROS‑dependent p38 MAPK signalling (Lu et al. 2018). Our findings also showed that ART could downregulate levels of pro-inflammatory factors in UC both *in vitro* and *in vivo*, which are in agreement with the previously reported data above.

Several studies have reported the role of miR-155 and NF-κB signalling in UC. It has been reported that miR-155 upregulated the expression of pro-inflammatory factor TNF-α, eventually leading to disease progression (Hou et al. 2017; Li et al. 2018). It is believed that TNF-α influences colitis related pathways like the NF-κB mediated signalling as well as recruitment and activation of immune cells (Béres et al. 2016). Furthermore, the activation of NF-κB signalling plays key roles to promote the progression of inflammation (Nunes et al. 2017) and downregulation of miR-155 contributes to inhibition of inflammation (Hou et al. 2017). Interestingly, our study findings are consistent with the previously reported results. We also found that inhibition of miR-155 could inhibit the expression of inflammatory factors and p-NF-κB induced by LPS, while overexpression of miR-155 showed opposite results and could reverse the therapeutic effects of ART.

Moreover, apoptosis is a predisposing factor for promoting inflammation which further worsens the degree of UC (Seidelin and Nielsen 2009; Lv et al. 2014). It was found TBNS could induce cell apoptosis and decrease cell viability of colonic cells through activation of NF-κB signalling in mice, which could be attenuated by paeoniflorin (Gu et al. 2017). Thus, it is reasonable to infer that blockade of apoptosis has the potential to improve UC. In our study, we showed that ART or downregulation of miR-155 inhibited LPS-induced apoptosis and increased cell viability, but overexpression of miR-155 could reverse the effects of ART. At the same time, ART treatment could inhibit miR-155 expression and NF-κB signalling pathway in mice, and reduced mice inflammatory response as well as improved mice survival rate. This result suggested that ART could inhibit the NF-κB signalling pathway by inhibiting miR-155, thereby improving cell viability and mice survival.

## Conclusions

This study found that ART treatment has the potential to alleviate UC by inhibiting the inflammatory response through suppressing the expression of miR-155/NF-κB axis. It may provide a new potential target for ART to treat UC.

## References

[CIT0001] Béres NJ, Szabó D, Kocsis D, Szűcs D, Kiss Z, Müller KE, Lendvai G, Kiss A, Arató A, Sziksz E, et al. 2016. Role of altered expression of miR-146a, miR-155, and miR-122 in pediatric patients with inflammatory bowel disease. Inflamm Bowel Dis. 22:327–335. 26752469 10.1097/MIB.0000000000000687

[CIT0002] Castelli AA, Estrada JJ, Kaminski JP. 2018. Patient with ulcerative colitis and abdominal pain. JAMA Surg. 153:282–283. 29299594 10.1001/jamasurg.2017.5521

[CIT0003] Chen YX, Zhang XQ, Yu CG, Huang SL, Xie Y, Dou XT, Liu WJ, Zou XP. 2019. Artesunate exerts protective effects against ulcerative colitis via suppressing Toll‑like receptor 4 and its downstream nuclear factor‑κB signaling pathways. Mol Med Rep. 20:1321–1332. 31173225 10.3892/mmr.2019.10345 PMC6625425

[CIT0004] Cooper HS, Murthy S, Shah R, Sedergran D. 1993. Clinicopathologic study of dextran sulfate sodium experimental murine colitis. Lab Inves. 69:238–249. 8350599

[CIT0005] da Silva BC, Lyra AC, Mendes CM, Ribeiro CP, Lisboa SR, de Souza MT, Portela RC, Santana GO. 2015. The demographic and clinical characteristics of ulcerative colitis in a northeast Brazilian population. Biomed Res Int. 2015:359130. 26509150 10.1155/2015/359130 PMC4609765

[CIT0006] Dondorp AM, Fanello CI, Hendriksen IC, Gomes E, Seni A, Chhaganlal KD, Bojang K, Olaosebikan R, Anunobi N, Maitland K, et al. 2010. Artesunate versus quinine in the treatment of severe falciparum malaria in African children (AQUAMAT): an open-label, randomised trial. Lancet. 376:1647–1657. 21062666 10.1016/S0140-6736(10)61924-1 PMC3033534

[CIT0007] Głąbska D, Guzek D, Zakrzewska P, Lech G. 2019. Intake of lutein and zeaxanthin as a possible factor influencing gastrointestinal symptoms in Caucasian individuals with ulcerative colitis in remission phase. JCM. 8:77–77. 10.3390/jcm8010077 PMC6352032 30641888

[CIT0008] Gu P, Zhu L, Liu Y, Zhang L, Liu J, Shen H. 2017. Protective effects of paeoniflorin on TNBS-induced ulcerative colitis through inhibiting NF-kappaB pathway and apoptosis in mice. Int Immunopharmacol. 50:152–160. 28666238 10.1016/j.intimp.2017.06.022

[CIT0009] Hou J, Hu X, Chen B, Chen X, Zhao L, Chen Z, Liu F, Liu Z. 2017. miR-155 targets Est-1 and induces ulcerative colitis via the IL-23/17/6-mediated Th17 pathway. Pathol Res Pract. 213:1289–1295. 28888763 10.1016/j.prp.2017.08.001

[CIT0010] Kalla R, Ventham N, Kennedy N, Quintana J, Nimmo E, Buck A, Satsangi J. 2015. MicroRNAs: new players in IBD. Gut. 64:504–513. 25475103 10.1136/gutjnl-2014-307891 PMC4345829

[CIT0011] Kim BJ, Yang SK, Kim JS, Jeen YT, Choi H, Han DS, Kim HJ, Kim WH, Kim JY, Chang DK. 2009. Trends of ulcerative colitis-associated colorectal cancer in Korea: a KASID study. J Gastroenterol Hepatol. 24:667–671. 19378391 10.1111/j.1440-1746.2008.05730.x

[CIT0012] Kim JW, Hwang SW, Park SH, Song TJ, Kim MH, Lee HS, Ye BD, Yang DH, Kim KJ, Byeon JS, et al. 2017. Clinical course of ulcerative colitis patients who develop acute pancreatitis. World J Gastroenterol. 23:3505–3512. 28596686 10.3748/wjg.v23.i19.3505 PMC5442086

[CIT0013] Kunte R, Kunwar R. 2011. WHO Guidelines for the treatment of malaria. Med J Armed Forces India. 67:376–376.

[CIT0014] Li T, Chen H, Yang Z, Liu XG, Zhang LM, Wang H. 2013. Evaluation of the immunosuppressive activity of artesunate *in vitro* and *in vivo*. Int Immunopharmacol. 16:306–312. 23583335 10.1016/j.intimp.2013.03.011

[CIT0015] Li J, Zhang J, Guo H, Yang S, Fan W, Ye N, Tian Z, Yu T, Ai G, Shen Z, et al. 2018. Critical role of alternative M2 skewing in miR-155 deletion-mediated protection of colitis. Front Immunol. 9:904–915. 29774026 10.3389/fimmu.2018.00904 PMC5943557

[CIT0016] Liao Y, Lei J, Liu M, Lin W, Hong D, Tuo Y, Jiang MH, Xia H, Wang M, Huang W, et al. 2016. Mesenchymal stromal cells mitigate experimental colitis via insulin-like growth factor binding protein 7-mediated immunosuppression. Mol Ther. 24:1860–1872. 27397633 10.1038/mt.2016.140 PMC5112041

[CIT0017] Li Y, Mu W, Xu B, Ren J, Wahafu T, Wuermanbieke S, Ma H, Gao H, Liu Y, Zhang K, et al. 2019. Artesunate, an anti-malaria agent, attenuates experimental osteoarthritis by inhibiting bone resorption and CD31hiEmcnhi vessel formation in subchondral bone. Front Pharmacol. 10:685. 31258481 10.3389/fphar.2019.00685 PMC6587439

[CIT0018] Livak KJ, Schmittgen TD. 2001. Analysis of relative gene expression data using real-time quantitative PCR and the 2^−ΔΔCT^ method. Methods. 25:402–408. 11846609 10.1006/meth.2001.1262

[CIT0019] Lu H, Wang B, Cui N, Zhang Y. 2018. Artesunate suppresses oxidative and inflammatory processes by activating Nrf2 and ROS‑dependent p38 MAPK and protects against cerebral ischemia‑reperfusion injury. Molr Med Rep. 17:6639–6646. 10.3892/mmr.2018.8666 29512760

[CIT0020] Lv B, Liu Z, Wang S, Liu F, Yang X, Hou J, Hou Z, Chen B. 2014. MiR-29a promotes intestinal epithelial apoptosis in ulcerative colitis by down-regulating Mcl-1. Int J Clin Exp Pathol. 7:8542–8552. 25674218 PMC4313986

[CIT0021] Min M, Peng L, Yang Y, Guo M, Wang W, Sun G. 2014. MicroRNA-155 is involved in the pathogenesis of ulcerative colitis by targeting FOXO3a. Inflamm Bowel Dis. 20:652–659. 24583476 10.1097/MIB.0000000000000009

[CIT0022] Nunes JJ, Pandey SK, Yadav A, Goel S, Ateeq B. 2017. Targeting NF-kappa B signaling by artesunate restores sensitivity of castrate-resistant prostate cancer cells to antiandrogens. Neoplasia. 19:333–345. 28319807 10.1016/j.neo.2017.02.002 PMC5358938

[CIT0023] Seidelin JB, Nielsen OH. 2009. Epithelial apoptosis: cause or consequence of ulcerative colitis? Scand J Gastroenterol. 44:1429–1434. 19958058 10.3109/00365520903301212

[CIT0024] Shanahan F. 1993. Pathogenesis of ulcerative colitis. The Lancet. 342:407–411. 10.1016/0140-6736(93)92818-e 8101906

[CIT0025] Singh UP, Murphy AE, Enos RT, Shamran HA, Singh NP, Guan H, Hegde VL, Fan D, Price RL, Taub DD, et al. 2014. miR-155 deficiency protects mice from experimental colitis by reducing T helper type 1/type 17 responses. Immunology. 143:478–489. 24891206 10.1111/imm.12328 PMC4212960

[CIT0026] Tian T, Zhou Y, Feng X, Ye S, Wang H, Wu W, Tan W, Yu C, Hu J, Zheng R, et al. 2016. MicroRNA-16 is putatively involved in the NF-κB pathway regulation in ulcerative colitis through adenosine A2a receptor (A2aAR) mRNA targeting. Sci Rep. 6:30824. 27476546 10.1038/srep30824 PMC4967855

[CIT0027] Tolstanova G, Khomenko T, Deng X, Szabo S, Sandor Z. 2010. New molecular mechanisms of the unexpectedly complex role of VEGF in ulcerative colitis. Biochem Biophys Res Commun. 399:613–616. 20682292 10.1016/j.bbrc.2010.07.124

[CIT0028] Xu XM, Zhang HJ. 2016. miRNAs as new molecular insights into inflammatory bowel disease: crucial regulators in autoimmunity and inflammation. World J Gastroenterol. 22:2206–2218. 26900285 10.3748/wjg.v22.i7.2206 PMC4734997

[CIT0029] Xue M, Shi L, Wang W, Chen S, Wang L. 2018. An overview of molecular profiles in ulcerative colitis-related cancer. Inflamm Bowel Dis. 24:1883–1894. 29945208 10.1093/ibd/izy221

[CIT0030] Yang Z, Ding J, Yang C, Gao Y, Li X, Chen X, Peng Y, Fang J, Xiao S. 2012. Immunomodulatory and anti-inflammatory properties of artesunate in experimental colitis. Curr Med Chem. 19:4541–4551. 22834815 10.2174/092986712803251575

[CIT0031] Zeng XZ, Zhang YY, Yang Q, Wang S, Zou BH, Tan YH, Zou M, Liu SW, Li XJ. 2019. Artesunate attenuates LPS-induced osteoclastogenesis by suppressing TLR4/TRAF6 and PLCγ1-Ca^2+^-NFATc1 signaling pathway. Acta Pharmacol Sin. 41:229–236. 31431733 10.1038/s41401-019-0289-6 PMC7468527

[CIT0032] Zhao K, Song Z. 1989. Distribution and excretion of artesunate in rats. Proc Chin Acad Med Sci Peking Union Med Coll. 4:186–188. 2631113

[CIT0033] Zhong W, Lu X, Shi H, Zhao G, Song Y, Wang Y, Zhang J, Jin Y, Wang S. 2017. Distinct microbial populations exist in the mucosa-associated microbiota of diarrhea predominant irritable bowel syndrome and ulcerative colitis. J Clin Gastroenterol. 53:660–672. 10.1097/MCG.0000000000000961 29210899

[CIT0034] Zuo S, Li Q, Liu X, Feng H, Chen Y. 2016. The potential therapeutic effects of artesunate on stroke and other central nervous system diseases. Biomed Res Int. 2016:1489050. 28116289 10.1155/2016/1489050 PMC5223005

